# Scar-Free Healing: Current Concepts and Future Perspectives

**DOI:** 10.3390/nano10112179

**Published:** 2020-10-31

**Authors:** Alexandra Elena Stoica, Alexandru Mihai Grumezescu, Anca Oana Hermenean, Ecaterina Andronescu, Bogdan Stefan Vasile

**Affiliations:** 1 Department of Science and Engineering of Oxide Materials and Nanomaterials, Faculty of Applied Chemistry and Materials Science, University Politehnica of Bucharest, 1–7 Gheorghe Polizu Street, 011061 Bucharest, Romania; elena_oprea_93@yahoo.co.uk (A.E.S.); grumezescu@yahoo.com (A.M.G.); ecaterina.andronescu@upb.ro (E.A.); 2 National Research Center for Micro and Nanomaterials, Faculty of Applied Chemistry and Materials Science, University Politehnica of Bucharest, 060042 Bucharest, Romania; 3 Institute of Life Sciences, Vasile Goldiş Western University of Arad, 310025 Arad, Romania; anca.hermenean@gmail.com

**Keywords:** wound healing, tissue regeneration, skin regeneration, scarring, scar-free wound regeneration, regenerative capacity, regenerative biomaterials

## Abstract

Every year, millions of people develop scars due to skin injuries after trauma, surgery, or skin burns. From the beginning of wound healing development, scar hyperplasia, and prolonged healing time in wound healing have been severe problems. Based on the difference between adult and fetal wound healing processes, many promising therapies have been developed to decrease scar formation in skin wounds. Currently, there is no good or reliable therapy to cure or prevent scar formation. This work briefly reviews the engineering methods of scarless wound healing, focusing on regenerative biomaterials and different cytokines, growth factors, and extracellular components in regenerative wound healing to minimize skin damage cell types, and scar formation.

## 1. Introduction

Every year, millions of people suffer from scars due to skin injuries after surgery, trauma, or burns, which have a significant physical and psychological impact [1,2]. Pathologic skin fibrosis causes scars that are mangled, reduce normal movement, and impede patient recovery and reintegration into society [3,4]. Scarring is a common consequence of skin injuries and can cause various adverse effects, including physical deformities and mental illness [1,5]. According to the World Health Organization (WHO) report, more than 11 million burns are reported to require medical care each year worldwide [1,6].

**Figure 1:**
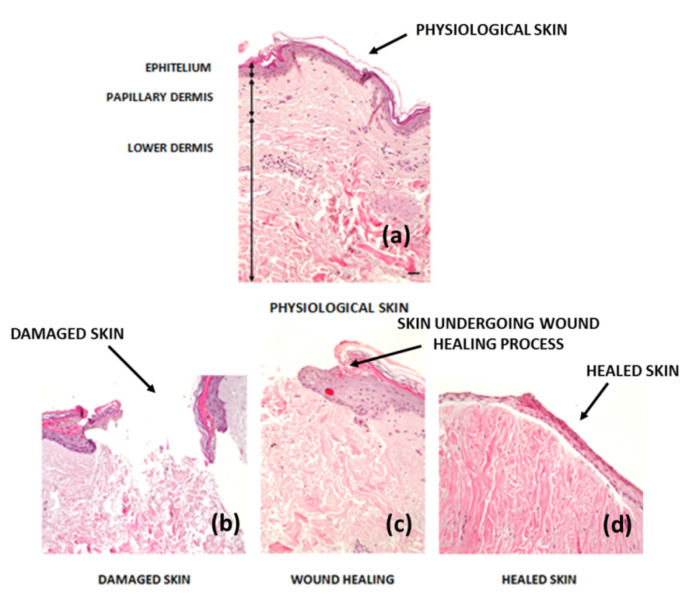
Histological image—hematoxylin–eosin stages of the wound healing process (10×, bar: 20 µm) (**a**) physiological skin, (**b**) damaged skin, (**c**) skin undergoing wound healing process and (**d**) healed skin [19].

Overproduction of connective tissue (especially collagen) in the time of wound healing may lead to scar formation (see Figure 1) [7,8]. Skin scar formation covers a variety of clinical phenotypes and could be divided into more subtypes. Bayat et al. [9] classified scars into “normal” fine line scars, a variety of abnormal scars, including widespread scars, atrophic scars, scar contractures, hypertrophic scars and keloid scars. If we discuss hypertrophic and keloid scars, both are produced by excessive deposition of immature collagen all along the remodeling phase [10]. Nonetheless, a hypertrophic scar forms a red raised mass in the initial wound site, which in some measures will shrink over time. Simultaneously, a keloid scar (a more severe form of significant scarring) will extend beyond the initial wound site with broad collagen bundles, which do not degenerate spontaneously [7,11].

In 1971, it was first shown that before the 120th day of pregnancy, the fetal lamb wound had healed without scarring [12]. Comparably, it is demonstrated that the skin damage of the human fetus in early pregnancy can be cured to develop normal skin tissue without scarring [13,14,15]. Engineering methods that mimic the fetal wound healing process can lead to techniques that minimize or erase scar generation after injury [1,16,17,18].

Although different treatments and therapies are used to target various wound healing stages to impede scar development, none of these treatments or therapies has been entirely successful [3,20,21]. This review presents an update on engineering strategies for scarless wound healing, focusing on the use of regenerative biomaterials, and treat the scope of various growth factors, cell types, cytokines, and extracellular components in regenerative wound healing. Combining the regenerative matrices based on biomaterials with engineered cells with the smaller intrinsic potential for fibrogenesis represents a promising approach for complete scar-free skin tissue regeneration.

## 2. Scar Healing Mechanism

The human skin has three layers: epidermis, dermis, and hypodermis. The epidermis is the outer layer, a barrier to the external environment, such as protecting the skin from ultraviolet (UV) radiation [22]. It contains layers of cells with regenerative abilities, so that superficial injuries, like scrapes, are apt to regenerate without scarring [23,24]. Beneath the epidermis is the dermis, which is as valuable a layer of the skin as the other two. The dermis contains an elaborate network of connective tissues, enhancing the elasticity and strength of human skin [25,26]. Hypodermis is an extremely viscous and soft layer [27]. Nonetheless, at the time of injury, these connective tissue networks are attacked, leading to injury-related scars. Skin scar formation includes normotrophic, hydrotropic, hypertrophic, contracture, and keloid scars. The overgrowth of collagen fibrous tissue causes these scars. In the process of rehabilitation, collagen fibrous tissue replaces normal skin. [26,28,29]. Hypertrophic scars are raised, unusually pigmented, and provoke itching or abnormal sensations [11,30]. If compared with keloid scars, hypertrophic scars remain within the line of actual damage and relapse over time [31,32]. They contain nodules, including myofibroblasts, which are differentiated fibroblasts representing alpha-smooth muscle actin, collagen fibers, and extracellular matrix (ECM) [28,29].

Even though considerable progress has been made in understanding the elaborate processes and networks that control human wound healing, regenerative medicine has still not been able to develop therapies that can induce the natural wound environment in order to heal without scars [33,34]. The lack of ability to induce perfect skin regeneration is somewhat due to our narrow understanding of scarless healing in the natural environment [35,36,37].

**Figure 2:**
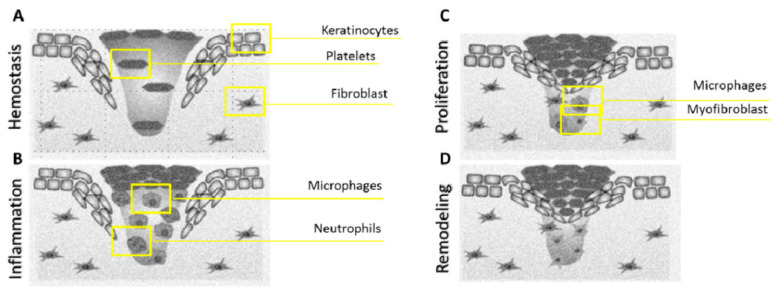
Wound healing process and cells involved in each phase: (**A**) hemostasis, (**B**) inflammation, (**C**) proliferation, (**D**) remodeling [19].

The healing process involves many closely coordinated regenerative reactions, counting hemostasis, the migration of various cell types to the wound, inflammation, angiogenesis, and the formation of extracellular matrix [1,2,38,39]. Skin wound healing in adults is well known and consists of four partially overlapping stages (see Figure 2): hemostasis, inflammation, proliferation, and remodeling. The last three stages determine whether the wound is healing naturally or whether the abnormal healing process causes excessive ECM protein production and fibrosis [2,40].

The extracellular matrix (ECM) forms a dynamic landscape of structural proteins and macromolecules that describes tissue structure and actively controls the form, behavior, and function of the cells [26,41,42,43]. In numerous situations, ECM provides cell polarity, barrier role, and defense for stem cell progenitor cells, all of which are vital for maintaining complex organisms [41,44]. Therefore, it is not strange that ECM plays a fundamental role in precisely regulated adult tissue repair and is a key element in determining the repair process’s efficacy and grade [41,45].

Scar formation is a key part of normal mammalian tissue regeneration, but its resolution defect may conduce to excessive accumulation of extracellular matrix (ECM) in the tissue and cause pathological scar formation. Additionally, excessive production of ECM components and tissue sclerosis are characteristic of other fibrotic conditions of the skin. Scleroderma affects a limited area of the skin, and systemic sclerosis affects the entire skin and internal organs. Fibrosis also disorders numerous other organs, like the heart, kidney, liver, and lungs, leading to acute dysfunction of these tissues [2,46].

The majority of adult skin injuries, the wound will form a disordered extracellular matrix that becomes a scar. However, the degree of occurrence is variable [47]. However, in some people, the result of wound healing is excessive accumulation of collagen, leading to hypertrophic scars or keloids [48]. Therefore, it can be said that scar formation is the final result of the repair process after tissue injury [49,50]. Thus, excessive scars seem to be determined by the following factors: (a) excessive production of collagen and different ECM components; (b) loss or downregulation of key stop signals; (c) dysfunction in the resolution and downregulation of the degradative enzymes implicated in eliminating the scar tissue [47].

About 4 days after inflammatory cells debride the wound, the damaged dermis begins to repair through granulation tissue formation. Under the stimulation of macrophages, fibroblasts and endothelial cells begin to migrate on the temporary wound matrix and enter the wound site with the support of MMPs [51]. Metalloproteinases (MMPs) are key enzymes implicated in ECM remodeling due to their capacity to degrade different matrix components, growth factors, cytokines and cell surface receptors. Finally, the fibrin clot is transformed into connective tissue rich in blood vessels, which becomes the basis for the granular appearance. This transition requires a balance between matrix degradation and production because insufficient ECM degradation or excessive production can lead to hypertrophic scar formation [52].

**Figure 3:**
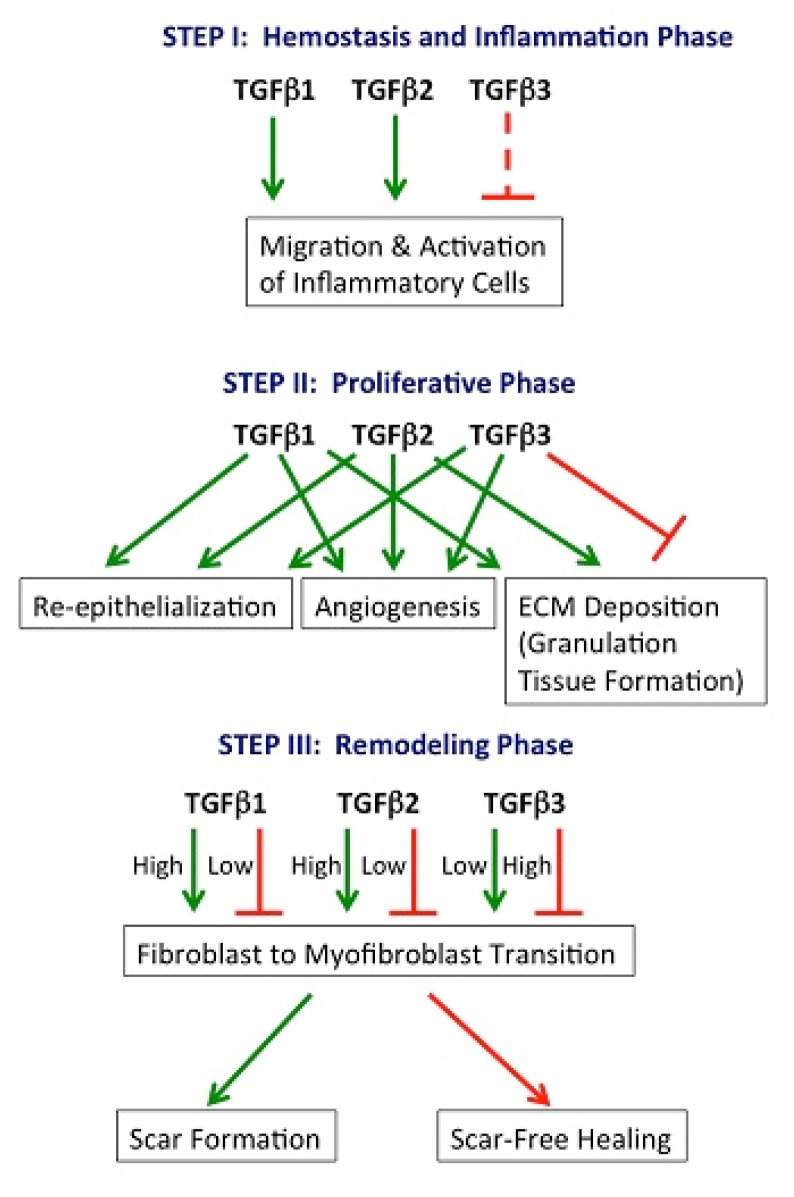
Transforming growth factor β (TGF-β) isoforms and their role in wound healing—schematical representation [54].

**Figure 4:**
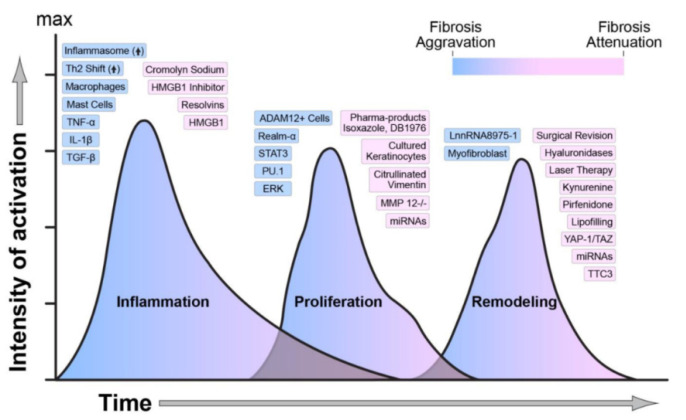
Regulators of wound healing and scarring (blue indicates activation, and pink indicates attenuation of fibrosis) [3].

The molecular mediators that regulate the wound healing stage are outlined in Figure 3 [3]. The core of wound repair is transforming growth factor β (TGF-β), a cytokine secreted by various cell types involved in healing [53]. TGF-β possesses three isoforms (TGF-β1, TGF-β2, and TGF-β3), all of which promote infiltration of inflammatory cells and fibroblasts and determine fibroblast proliferation, angiogenesis, and also ECM production (see Figure 4). In addition, TGF-β inhibits reepithelialization [52,54].

Another suggestive growth factor in the pathogenesis of hypertrophic scars and other fibrotic diseases is the connective tissue growth factor (CTGF). As a mediator of various effects of TGF-β1 on fibroblasts, CTGF supports the production of ECM, counting type I collagen and fibronectin. After all, CTGF can regulate MMPs and their inhibitors (TIMPs), consequently regulating the stability and integrity of the ECM. So, it appears that CTGF is valuable for granulation tissue and subsequent scar formation [52,55].

In humans, there is one exception to this healing process. In the first trimester of pregnancy, the fetus heals with little or no scar formation [49,50]. Human fetal skin in early pregnancy can regenerate after injury without forming scar tissue. Fetal wound healing involves many factors, including cytokines, soluble growth factors, and insoluble extracellular matrix (ECM) proteins and tissue mechanics. Recent studies on adult wound healing (considering the fetal wound healing model) have shown that only a single factor (like transforming growth factor-β3 (TGF-β3) or interleukin-10 (IL-10)) cannot achieve efficient scar-free wound healing. Research centered on the fetal regeneration process has shown that scarless wound healing can be achieved by combining various cells and morphogenetic factors within a fetal–mimetic matrix or scaffold [1,56,57,58]. Understanding the differences between fetal and adult skin wound healing may conduct new and efficient scar-free skin regenerative therapies.

## 3. Current Scar Treatments and Involved Factors

Although many treatments have been used for various scars, none of them has gained satisfactory clinical results. Over time, cryotherapy, steroid infusion, weight therapy, and medication have been used for skin regeneration, but patient satisfaction is low. Therefore, scars are still a challenge in the cosmetic and surgical area [4,28,59].

In order to develop new drugs for the prevention and removal of scars, researchers focused on the results of scarless skin regeneration models, such as human fetuses [60,61,62], American black bear [63], reindeer [28], African spiny mouse [64,65], bottlenose dolphin [66], fish-scale gecko [67], zebrafish [68,69,70], salamander [71,72] and amphibians [73,74,75], which, using endogenous stem cells to prevent scar formation, shows significant regeneration and repair capabilities during wound healing. Therefore, we focus here on the engineering methods of scarless wound healing, focusing on the use of regenerative biomaterials, advanced therapeutic dressings, and different growth factors, cytokines and extracellular components for scar-free wound healing [28].

### 3.1. Cellular Factors Mediating Scar-Free Healing

**Figure 5:**
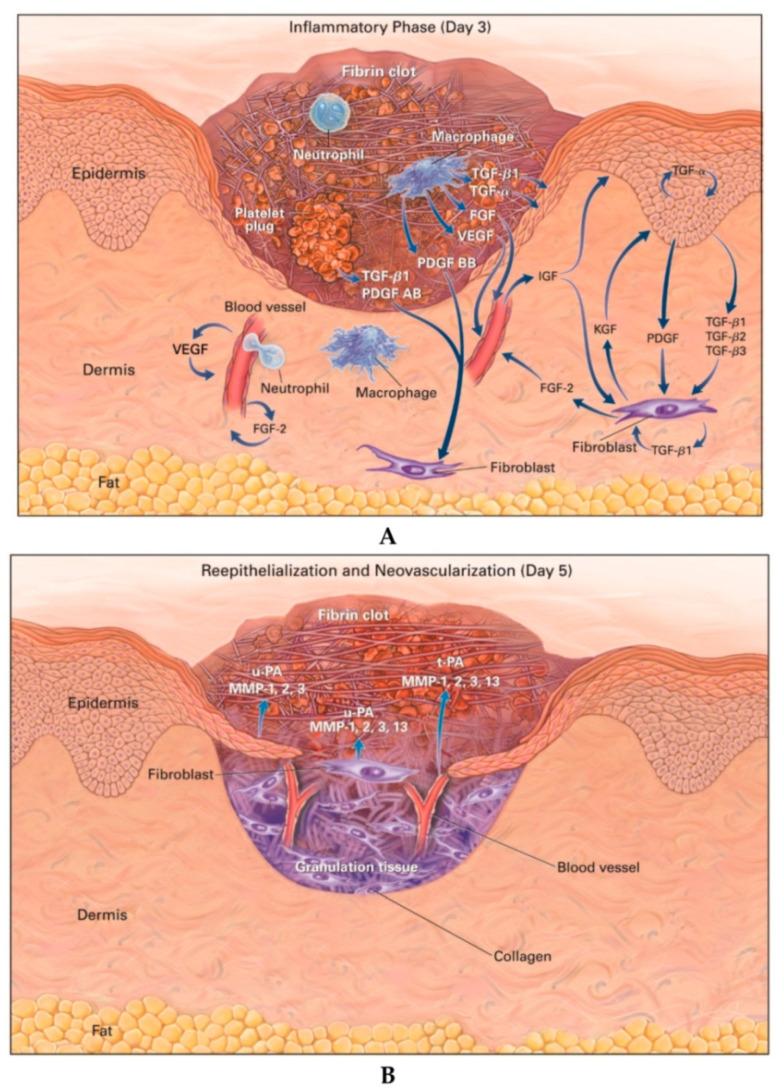
Cutaneous wounds (**A**) 3 days and (**B**) 5 days after injury [7].

Wound healing is a very complex process affected by many factors. In the stage of rehabilitation, a distinct set of specific growth factors and cytokines must interact with their receptors, different growth factors, and extracellular matrix (ECM) components at their target sites [7,76]. The wound healing sequence that leads to tissue scars starts with the local inflammation in the tissue; the actual trauma is not mandatory and can be replaced by other stimuli and cascade motion (see Figure 5) [28,33,49].

Skin damage exposes platelets from blood vessels to subendothelial collagen, conducting to thrombin formation. Platelets activated by thrombin release some growth factors, finally forming a hemostatic plug [7,77]. Growth factors represent endogenous signaling molecules that can control cellular responses necessary for wound healing mechanisms (such as migration, proliferation, and differentiation) [7,78]. Growth factors released from activated platelets include heparin-binding EGF-like growth factor (HB-EGF), epidermal growth factor (EGF), insulin-like growth factor 1 (IGF-1), platelet-derived growth factor (PDGF), platelet-derived endothelial cell growth factor, transforming growth factor (TGF)-alpha and TGF-beta (TGF-β) [7,28]. These growth factors diffuse into surrounding tissues and chemotactically attract monocytes and neutrophils into the wound [7,79]. Neutrophils are the most frequent inflammatory cells at the early stages of the wound healing process. These cells are vital to the wound-healing response by mobilizing other inflammatory cells, like monocyte monocyte-derived macrophages, phagocytosing the fibrin clot, and removing debris of tissue and also dead cells [49,80]. Neutrophils reach the wound site and eliminate microorganisms within the wound bed through phagocytosis and the delivery of antimicrobial granules. Neutrophils also have the role to release chemokines that bring more leukocytes in order to increase the immune response [81,82].

The proliferation phase is defined by granulation tissue formation and the beginning of angiogenesis [7,83]. Granulation tissue consists of neovasculature, fibroblasts, macrophages in a free collagen matrix, hyaluronic acid (HA), and fibronectin. It finally replenishes the wound area. At this stage, the number of inflammatory cells decreases, platelet-derived growth factor (PDGF) and transforming growth factor β (TGF-β) released from inflammatory cells chemotactically bring fibroblasts into the wound site [7,84,85].

**Figure 6:**
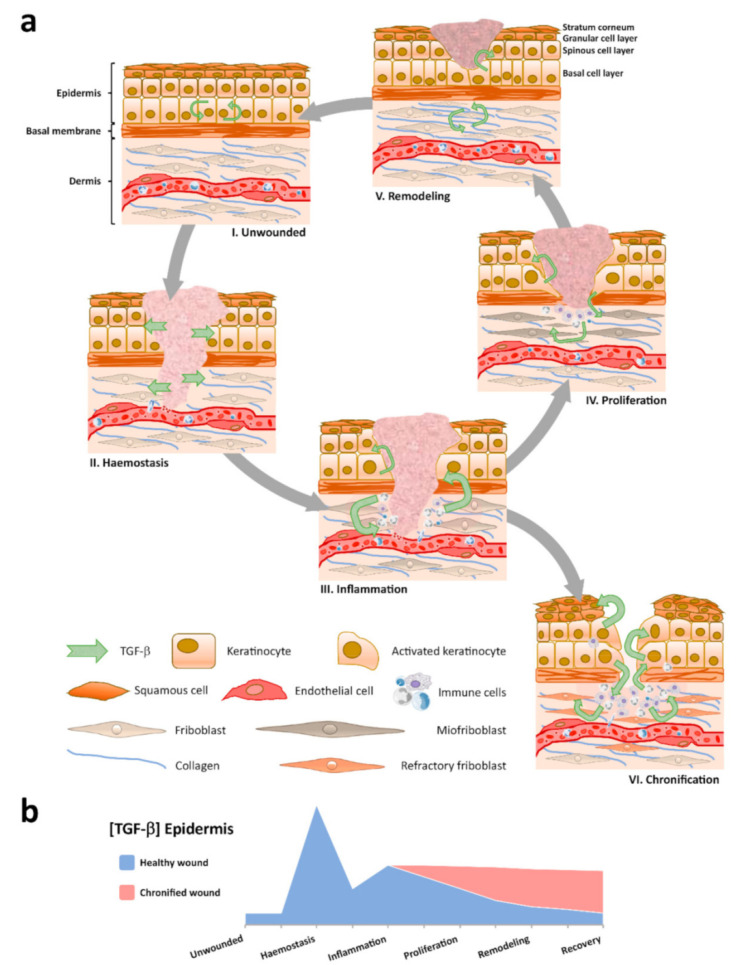
(**a**) Various TGF-β signatures; (**b**) the amount of TGF-β varies in the epidermis through the sequential stages of wound healing [89].

The particular binding of growth factors to their receptors activates intracellular signal transduction pathways, thereby regulating different aspects of subcellular physiology and cell function [7,86]. Growth factors link to their equivalent receptors placed on the cell surface, opening signaling pathways to activate important signaling molecules that may stimulate cytoplasmic proteins or lead transcription of novel proteins [7,87]. The role of some fibrogenic signaling molecules, counting TGF-β (see Figure 6), IL-4, IL-6, IL-8, IL-10 and IL-13, TNF-α, IGF-1, KGF, PDGF, EGF, VEGF, CTGF, FGF-1, FGF-2 and plasminogen activator inhibitor 1 (PAI1), have been well documented [28,49,88].

Thus, we can declare that diverse signaling pathways and GFs (see Table 1) are implicated in fibrotic pathologies’ genesis, and they are often intertwined. This makes it more challenging to develop new therapies because a single upstream element that regulates these signaling networks can significantly impact other cellular mechanisms [49,90,91,92].

Therefore, as understanding the function of growth factors in the pathophysiology of chronic wounds improves, growth factors are expected to provide the best wound treatment method. Nonetheless, they have restricted clinical applications due to the short in vivo half-life as a result of their low stability, limited absorption through the skin around wound injury, elimination by exudation before achieving the wound site, and unwanted results due to high local and/or systemic levels after topical administration [7,104].

### 3.2. Advanced Therapeutic Dressings for Scar-Free Wound Healing

Wound healing is a process common to all vertebrates [105]. Many scar-free techniques, materials, and treatments have been developed over time. For clinical and preclinical treatments a variety of vertebrates, including amphibians [35,106,107], fish [69], reptiles [95], and mammals (counting mice, marsupials, sheep, rats, rabbits, pigs, and frogs) [105,108] have been used [105,109]. Wound healing ranges from non-amniotic vertebrates’ ability to completely regenerate tissues to the formation of skin scars in adult mammals and humans [105].

The latest developments in wound repair and regeneration have promoted the use of different bioresource-based nanocomposite dressings. These dressings may solve the problems related to conventional dressings, which can cause tissue damage and cannot effectively prevent infection [110].

**Figure 7:**
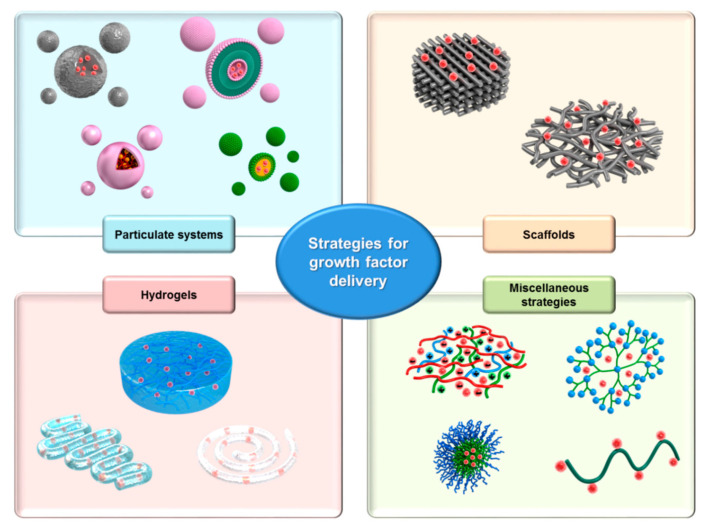
Growth factor-loaded drug delivery systems (DDSs) for enhanced wound healing [7].

Obtaining a valuable nanocomposite system consists of natural polymers regarding drug incorporation and drug release, representing a green approach working at the fusion of nanotechnology and biomedical science [110,111,112]. Further, various drug delivery systems (DDSs) have been developed to assure better stability and controlled release of growth factors for the treatment of acute and chronic wounds (see Figure 7) [7,113,114].

Therefore, the focus of research has been on the application of naturally derived biopolymers like dextran [110,115] and chitosan [116,117,118] based on their biodegradability, biocompatibility, ease of availability, and chemical transformation that decrease their biological properties in terms of therapeutic outputs [110,119,120]. One such example was published by Zhang et al. [116] in a study aimed to determine if and how the cationic of chitosan can affect the hypertrophic scar-related outcomes through preparing carboxymethyl chitosan hydrogels with various genipin concentrations (2.5%, 5%, 10%, and 15%, respectively). Moreover, the authors proved that CMCS-5% sample with additional incorporation of 2% (*V*/*V*) Aloe vera gel presented a further improved scar prevention performance. Overall, these findings may have important implications for high-quality wound repair and effective scar suppression in the design of chitosan-based wound dressings.

Functionalized wound dressing has been demonstrated to increase wound healing, angiogenesis, and control newly formed types of collagen. It enhances the structure of already formed collagen to decrease its volume around the injured site [49]. Combined with electrospinning technologies, which utilize electrical forces to produce fibrous scaffolds on the nanometer to micrometer scale with controlled porosity, a type of advanced healing dressing should be obtained [121,122]. These scaffolds may be efficient due to their ability to mimic the natural ECM structure, assuring mechanical strength, supporting cell adhesion and proliferation, avoiding dehydration and supporting coverage, conducing to tissue repair in vivo. Their porous structure can also control the loading and release of drugs at the injury site, thereby increasing their antifibrotic compounds [49]. One such example is seen in a study inspired by fetal scarless wound healing. Dongmei Zhang et al. [123] developed a wearable biomimetic film (WBMF) obtained from hyaluronan (HA), vitamin E (VE), dopamine (DA), and β-cyclodextrin (β-CD) intending to mimic the fetal context (FC) and fetal extracellular matrix (ECM) around the wound site for dermal regeneration. The authors proved that the WBMF treatment of the severe full-thickness skin defect of adult mice, mimicking fetal ECM, could promote fibroblast migration, angiogenesis, reepithelization, and granulation tissue formation. More importantly, the WBMF could reduce collagen synthesis, reestablish typical dermal collagen architecture, and recover scarless appearance. Another biomimetic electrospun nano-fibrous antimicrobial dressing material loaded with dual antioxidants has been prepared to solve infections and scar formation [124]. Pandey et al. [124] developed a novel composite nano-fibrous material (PVP-Ce-Cur NF) composed of polyvinyl pyrrolidone (PVP), cerium nitrate hexahydrate (Ce(NO_3_)_3_·6H_2_O), and curcumin using an electrospinning technique. So, the PVP-Ce-Cur NF dressing was directly applied to the full-thickness circular excision wound of Wistar model rats. It showed complete healing and re-epithelialization within 20 days, without any scars.

Encouraged by the fundamental roles of decorin (a small dermatan sulfate proteoglycan that can build extracellular matrix in collagen-containing tissues [125]), Jeon et al. [126] prepared an anti-scar collagen-targeting glue, which consists of newly designed collagen combined with mussel adhesive protein and specific glycosaminoglycans. In a rat skin resection model, the collagen-targeting glue successfully accelerated the wound’s initial regeneration, which was defined by effective re-epithelialization, new blood vessel formation, and rapid collagen synthesis.

A study represents another example of wound dressing inspired this time by the wound healing characteristics of the oral mucosa [83,127]. In short, there are many biological differences between the oral mucosa and other skin tissues, one of which is the expression of cytokines. The biological performance of oral mucosal epidermis in rapid healing after trauma also proves the high expression of cytokines related to epidermal growth and proliferation [128]. Even if the cytokines responsible for cellular proliferation and migration have different mechanisms, their role in promoting wound healing is beyond doubt. Over time, the oral mucosa has formed a highly antibacterial, anti-inflammatory, and moist tissue environment that the skin does not have. This is another huge difference between the two tissues [83,127]. A biomimetic hydrogel was prepared by Kong et al. [83] to realize the rapid and scar-free healing of skin wounds. The bFGF, EGF, lysozyme, and HA were selected as the essential components that are differently expressed between the oral mucosa and the back skin. By embedding exogenous EGF into layered self-assembled microcapsules and embedding bFGF into hydrogels, the differential release effects of bFGF and EGF similar to those of the oral mucosa were tactically controlled. They concluded that the sequential expression of simulated cytokines and the sterile, moist environment of the oral mucosa are useful and necessary for rapid and scarless skin healing.

Zinc is essential for humans and is a cofactor for many transcription factors and enzymes, which play a vital role in growth, metabolism, immune function, and wound regeneration [129,130]. Metal nanoparticles, such as zinc, are generally considered safe in wound healing and burn treatment due to their low toxicity, antibacterial, bactericidal and anti-inflammatory properties [129,131,132]. In this context, zinc sulfide (ZnS) nanoparticles were combined with a transglutaminase cross-linked gelatin gel (Col-Tgel) carrier and delivered in a rodent wound bed model [129]. Therefore, Han et al. [129] investigated zinc sulfide nanoparticles (ZnS-NP) on wound healing in vitro with 2D and 3D models and in vivo with a rat full-thickness wound model. Wounds treated with ZnS nanoparticles promoted skin regeneration by inducing skin appendage formation, dermal papillae, and adipocyte migration. ZnS treated group inhibited wound contraction and scar tissue formation.

Adipose-derived stem cells (ASCs, represent a rich source of mesenchymal stem cells (MSC)) can be obtained from subcutaneous fat tissue through minimally invasive procedures. ASC has shown great potential in many cell-based therapies. Their ability to repair and regenerate skin wounds is most likely to be mediated through differentiation and paracrine effects [133,134]. Adipose-derived stem cell (ASC) sheets may be obtained easily using stimulation with l-ascorbate 2-phosphate and have important tissue regeneration applications and wound healing [133,135]. Recently, ASCs have been shown to have therapeutic effects on fibrosis and difficult scars. Yu et al. [133] fabricated L-ascorbate 2-phosphate (A2-P) induced ASC sheets that proved physiological cues to enhance skin wound healing and reduce scar formation, thus representing an effective topical wound treatment modality.

**Figure 8:**
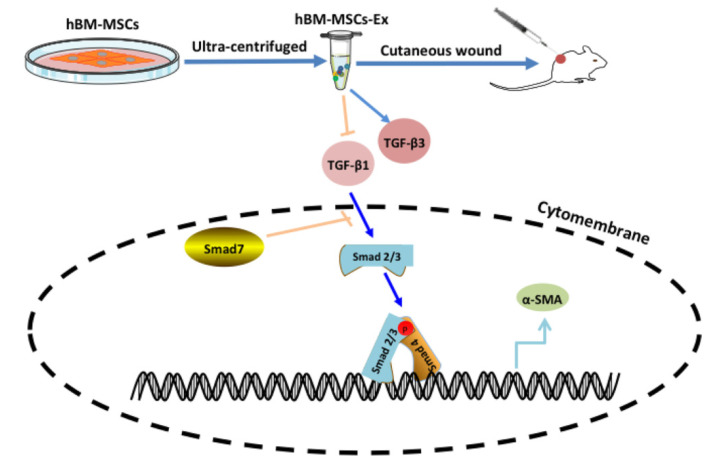
Schematically ilustration of hBM-MSC-Ex stimulates cutaneous wound healing by regulating the TGF-β/Smad signal pathway [138].

The mesenchymal stromal cells (MSC) have immunomodulatory characteristics and a high regenerative ability. The MSC were first reported as presenting a similar morphology to fibroblasts. The MSC have been isolated from various tissues, including fatty tissue, bone marrow, synovium, hair follicle, dental pulp, Wharton’s jelly, placenta, umbilical cord, umbilical cord blood and periodontal ligament. In addition, MSC-derived exosomes may be a great alternative to MSC cell therapy as they possess similar biological functions to the originating cells. Moreover, MSC exosomes are more stable and have lower immunogenicity compared to their originating cells [136,137]. Jiang et al. [138] studied the cell regeneration effects and its underlying mechanism of human bone marrow mesenchymal stem cell-derived exosomes (hBM-MSC-Ex) on cutaneous wound healing in rats (see Figure 8). Their results proved that hBM-MSC-Ex effectively promote the cutaneous wound healing through inhibiting the TGF-β/Smad signal pathway.

In addition, in 2018, Sun et al. [139] showed that Wharton’s jelly-derived mesenchymal stem cells (WJ-MSCs) could be a promising candidate for the therapy of cutaneous wounds. They investigated whether a WJ-MSC-derived conditioned medium (MSC-CM) may be used to cure radiation-induced skin wounds in rats. The authors proved that MSC-CM promoted cell proliferation, sebaceous gland cell-like regeneration and angiogenesis, accelerated wound closure and enhanced the quality of wound healing. Currently, some animal models are used to study scar formation (rabbit, rats and mice mostly). However, apart from the ethical issues, there is one big limitation: the physiology of animal model skin and their immune systems are very different from humans to the extent that pivotal factors responsible for differences between normotrophic, hypertrophic and keloid scar formation are hard to identify [140].

The new studies reviewed here shed light on the cellular and molecular mechanisms leading to scarring and regeneration upon skin injury. The current situation is continuously updated through multiple efforts in different fields, including interdisciplinary science, materials science engineering, biomedical engineering, and gene therapy, to name a few.

## 4. Conclusions

Skin wound repair is a versatile process designed to achieve two main tasks: restore the skin’s barrier function to avoid further blood loss and/or infection, and restore mechanical and also physiological properties.

Growth factors are natural peptides implicated in cell growth, proliferation, migration, and differentiation. The particular binding of different growth factors to their receptors activates intracellular signal transduction pathways that control different features of subcellular physiology and cell function. Many scientific researchers have proved that regulating the release of growth factors from pharmaceutical preparations by using DDS-based strategies may improve the wound healing process and skin regeneration.

Due to the heterogeneity under different fibrotic conditions, individual elements’ regulation seems insufficient in most cases; many current therapies have failed to obtain clinically relevant results. Although many advances have been made in the treatment of skin wounds, including different types of wound dressings, cell-based strategies, growth factors, and vacuum-assisted treatment, none of them can support scarless wound healing in adult skin. Therefore, it is essential to shift the target of new treatment methods from single-target modulation therapy to multi-target modulation therapy to maximize synergy and reduce harmful side effects.

Understanding the primary mechanism of scar formation and regeneration balance is the key to achieving scarless regeneration in a controlled manner in the future.

## Figures and Tables

**Table 1:** Cytokines and growth factor (GF) of current therapeutic importance in the modulation of wound repair and scarring [93].

Name	Predominant Cellular Source	References
platelet-derived growth factor (PDGF)	Platelets and macrophages	[1,94]
transforming growth factor β-1 (TGFβ-1), transforming growth factor β-2 (TGFβ-2)	Platelets and macrophages	[54,95]
transforming growth factor β-3 (TGFβ-3)	Fibroblasts and Keratinocytes	[54,96]
fibroblast growth factor-1 (FGF-1/aFGF), fibroblast growth factor-2 (FGF-2/bFGF)	Macrophages, fibroblasts and endothelial cells	[95,97]
keratinocyte growth factor (KGF)	fibroblasts and endothelial cells	[1]
epidermal growth factor (EGF)	Macrophages and Keratinocytes	[94,98]
vascular endothelial growth factor (VEGF)	Keratinocytes	[95]
interferon-gamma (IFNγ)	T Cells	[99,100]
insulin-like growth factor (IGF)	Platelets, Macrophages, Fibroblasts	[1]
tumor necrosis factor alpha (TNFα)	Macrophages	[1]
interleukin-1β (IL-1β)	-	[101]
interleukin-6 (IL-6)	Macrophages and Fibroblasts	[13,101,102]
interleukin-8 (IL-8)	Macrophages and Fibroblasts	[102,103]
interleukin-4 (IL-4)	T Cells	[99,100]
interleukin-10 (IL-10)	Macrophages and T Cells	[99,100]
